# Eye metastasis in breast cancer: case report and review of literature

**DOI:** 10.3332/ecancer.2022.1353

**Published:** 2022-02-10

**Authors:** Ereny Samwel Poles Saad, HebatAllah Mahmoud Bakri, Amal Rayan, Dina Barakat, Mariam Mohsen Khalel

**Affiliations:** Department of Clinical Oncology, Faculty of Medicine, Assiut University, Kornish Al Ibrahimeya, Asyut Second, Assiut Governorate, Assiut 71515, Egypt

**Keywords:** breast cancer, eye metastases, treatment, chemotherapy, radiotherapy

## Abstract

The paradigm of breast cancer management has been revolutionised, resulting in prolonged survival that echoes an increasing incidence of metastasis in uncommon sites. With orbital metastases – despite being rare – the incidence scales up to 13% of breast cancer cases with no specific management guidelines. We report a case of a 31-year-old luminal B breast cancer patient who initially presented with T2N2M0 disease and received six cycles of adjuvant chemotherapy (5-Fluorouracil (5-FU) 600 mg/m^2^ IV, Doxorubicin 60 mg/m^2^ IV, Cyclophosphamide 600 mg/m^2^ IV), followed by radiotherapy (RTH) and adjuvant Tamoxifen. Two years later, the patient experienced successive bone metastasis, so she received several lines of endocrine therapy as Fulvestrant and aromatase inhibitors in combination with luteinizing hormone-releasing hormone (LHRH) analogues. Later on, she presented with right eye ptosis and magnetic resonance imaging (MRI) showed a soft tissue mass in the superior and lateral rectus muscles. The patient received six cycles of chemotherapy with no improvement. Further disease progression occurred 3 months later, so the patient received palliative RTH resulting in no response. One month later, the patient was deceased, secondary to progressive disease. With the rising incidence of ocular metastasis due to breast cancer, oncologists should be aware of symptoms and use the proper diagnostic modalities. Here we provide a literature review on similar cases and discuss possible treatment modalities for those patients. The main concern is to evaluate the need for chemotherapy in such cases in the presence of highly effective endocrinal treatment.

## Introduction

Eye metastases are a rare event in cancer patients with breast cancer being the most common primary site (28.5%–58.8%) [1]. The rising incidence of eye metastasis of breast cancer origin can be attributed to the recent advances in the systemic treatment of breast cancer which has resulted in prolonged survival of breast cancer patients in addition to the improvements in diagnostic modalities [2, 3]. In most cases, eye metastasis occurs along with systemic progression of previously diagnosed breast cancer; however, 25% of diagnosed eye metastases are detected in patients with *de novo* breast cancer as an initial presentation [4].

The aim of this literature review is to demonstrate different treatment modalities in cases described in the literature to help to evaluate best treatment options in addition to explaining our local experience with a case of breast cancer with eye metastasis in terms of the challenges in treatment based on the limited resources available.

### Patient information, clinical finding, diagnostic assessment

**Figure 1.**
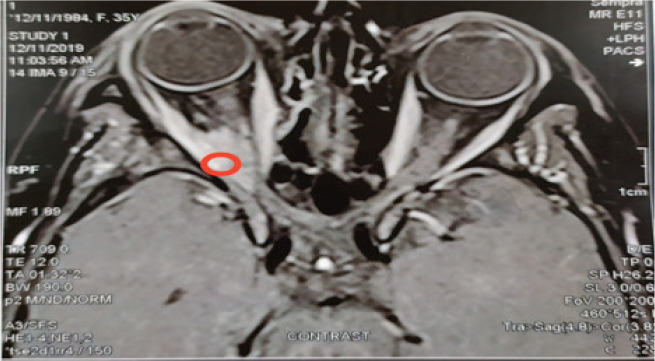
MRI brain at dignosis: T1 with contrast shows right superior rectal muscle thickening about 14 mm associated with slight proptosis of the right eye.

We report a case of a 31-year-old patient who was diagnosed with T2N2M0 Estrogen Receptor(ER)/Progesterone (PgR) positive/HER-2/neu negative, Ki-67 > 30% right breast cancer. She was treated with modified radical mastectomy followed by adjuvant chemotherapy with six cycles of FAC regimen (5-Fluorouracil (5-FU) 600 mg/m^2^ IV, Doxorubicin 60 mg/m^2^ IV, Cyclophosphamide 600 mg/m^2^ IV) every 21 days, radiotherapy (RTH) and adjuvant hormonal treatment with Tamoxifen for 2 years. The patient presented with severe back pain and the bone scan showed multiple bone metastases while multi-slice computed tomography (MSCT) of the chest and pelvis-abdomen were insignificant so she was shifted to luteinizing hormone-releasing hormone (LHRH) analogues in combination with Fulvestrant and palliative RTH. Eleven months later, the patient experienced successive bone progression and thereafter was shifted to aromatase inhibitors. Six months later, on November 2019 (4 years after the initial diagnosis), the patient presented with right eye ptosis, and the magnetic resonance imaging (MRI) of the brain and orbit showed a soft tissue mass in the superior and lateral rectus muscles (Figure 1) with no evidence of visceral metastasis on further metastatic work up except for multiple bone lesions.

### Therapeutic intervention and outcome of treatment

**Figure 2.**
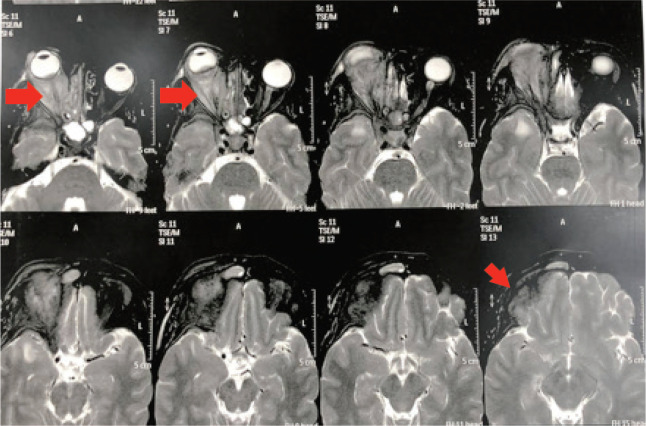
MRI brain and orbit showed significant increase in the previously described right recti muscles thickness with heterogeneous post-contrast enhancement. There is subsequent proptosis and posterior ocular coat mass lesion just above the optic disc with significant enhancement of all posterior ocular coats. There is extra-orbital spread, and intra-cranial extra-axial leptomeningeal enhancement at the right frontotemporal region.

**Figure 3.**
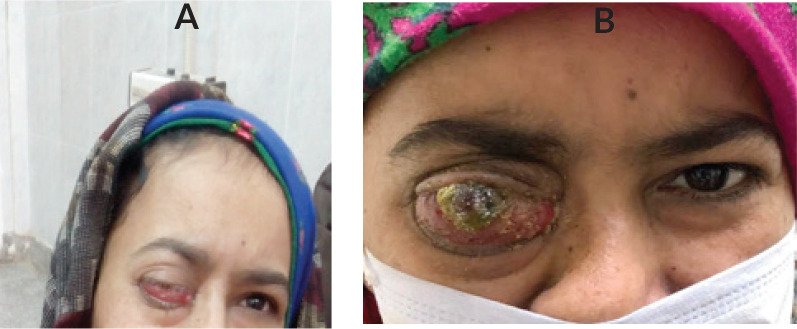
The presentation of the patient after the end of chemotherapy (a) and on progression after the radiotherapy (b).

After a multidisciplinary discussion, she started a combination chemotherapy of cisplatin and gemcitabine with a stationary course followed by Anastrozole. Three months later, the patient experienced further progression of her symptoms, in the form of ulceration, severe pain and pus discharge. The patient received palliative RTH at the dose of 30 Gy in ten fractions with no response (Figures 2 and 3). The patient was deceased 1 month later upon the deterioration of the general condition and further disease progression.

## Discussion

Metastatic carcinoma of the eye is an uncommon clinical situation, and the most prevalent primary tumour is breast carcinoma which accounts for 28.5%–58.8% of all orbital metastases [3] followed by lung cancer (24%) and skin melanoma (14%) [1]. The increased incidence of eye metastasis of breast cancer follows the advances in diagnostic modalities and the prolonged survival of breast cancer patients. MRI remains the gold standard diagnostic imaging modality [5].

**Figure 4.**
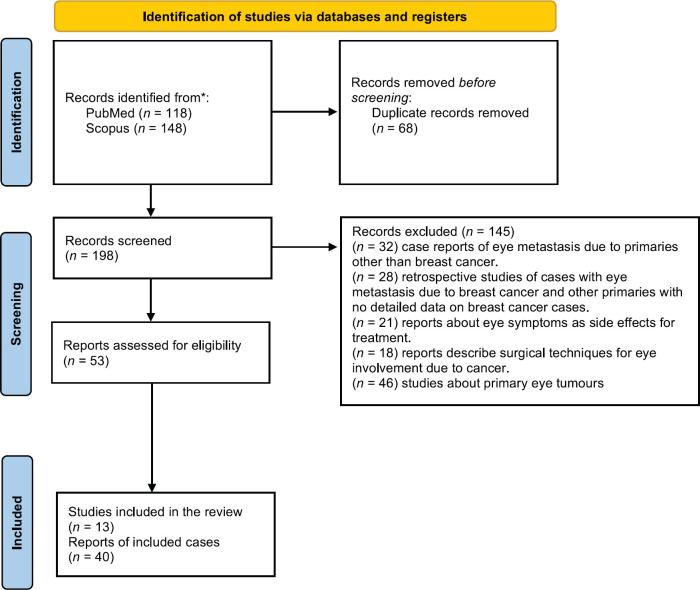
PRISMA flow diagram. From: Page MJ, McKenzie JE, Bossuyt PM, Boutron I, Hoffmann TC, Mulrow CD, et al. The PRISMA 2020 statement: an updated guideline for reporting systematic reviews. BMJ 2021;372:n71. doi: 10.1136/bmj.n71. For more information, visit: http://www.prisma-statement.org/

A PubMed and Scopus search including English language only was performed using the Med search words ‘breast cancer’, ‘eye metastasis’ and/or ‘orbital metastasis’ until January 2021. The literature search revealed 53 records, of which 13 were included in the review and 40 in the quantitative analysis (Figure 4) with 94 cases collectively which were included in the analysis (Supplementary Table 1).

Eye metastases secondary breast cancer may be presented at any time point of the course of the disease, 38 (40.4%) of cases included in the analysis presented with eye metastases as the initial presentation of breast cancer, while 56 (59.5%) developed eye metastases either as the only site of metastasis or as a part of the systemic progression of previously diagnosed breast cancer. In those patients, the time interval between the diagnosis of breast cancer and the development of eye metastasis when reported had a wide range from 1 month [6] up to 25 years [7], and 13 cases developed eye metastasis within 5 years of being diagnosed with early breast cancer. In another review, Freedman *et al* [8] reviewed the charts of 112 patients (141 eyes) and showed that the average time was approximately 4 years (1,266 days) from the breast cancer diagnosis to the occurrence of metastasis to the eye and orbit.

**Figure 5.**
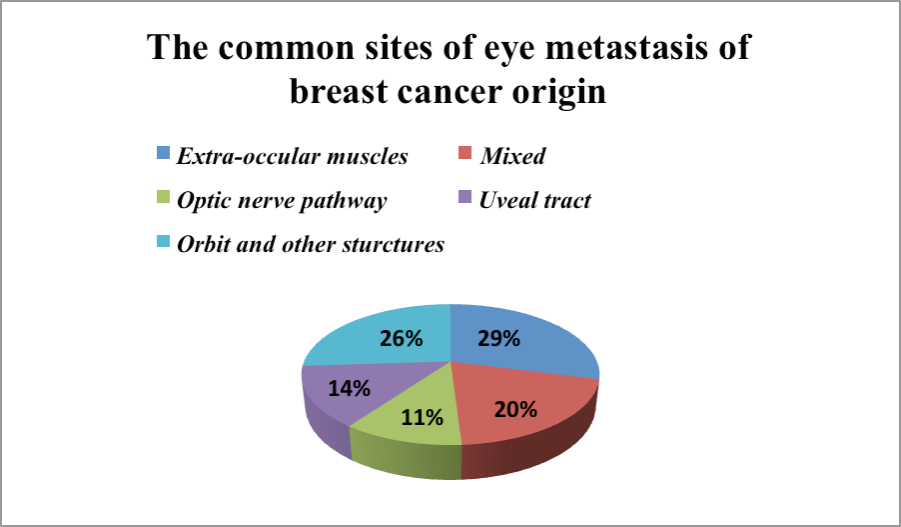
The most common structures within the eye with high affinity to metastasis from breast cancer.

Evaluation of the most common sites of the eye to be affected with metastasis of breast cancer was available for 66 cases as there was an overlap of data presented in one report as shown in Figure 5 [9]. There is controversy about the affinity of the breast cancer cell to specific tissue types within the eye; despite the extra-ocular muscles are rare to be affected [10] – based on the fact that the constant movement of muscles would prevent lodging of neoplastic cells [11] – but they were involved in one-third of the reviewed cases. Orbital involvement with annexes like the lacrimal gland was the second common site followed by the infiltrative mixed lesions that could affect more than one definitive structure. The uveal tract involvement was infrequent when compared with previous reports [12, 13].

The infiltrative ductal carcinoma (IDC) represented only half of the cases settling with one-third of patients with infiltrative lobular carcinoma (ILC); that is relatively higher than the prevalence of lobular carcinoma in the general breast cancer population [14]. The infiltrative nature of ILC could explain this discordance; Raap *et al* [15] reported that orbital metastases were attributed five times to ILC more often than to IDC. The luminal breast cancer subtype relates to the highest risk of eye metastasis compared with other aggressive subtypes like triple-negative breast cancer [16]. In two cases, the metastatic lesion in the eye turned ER/PR negative in primary hormone-positive breast cancer patients [9, 17] (Table 1).

There was a wide range of treatment modalities; mono-therapy or multimodality therapy with variable response outcomes (Table 1). Luminal breast cancer constituted the majority of cases, so we were concerned with treatment options used in hormonal positive breast cancer cases with eye metastasis. The insisting question is whether to consider eye metastasis as a visceral crisis that indicates chemotherapy or tumour progression to shift to other lines of hormonal treatment according to the guidelines [18]. Patients treated 10 years back were shifted from hormonal to chemotherapy when presented with eye metastasis resulting in a modest symptoms improvement with no available survival data [4, 5]. Reports published in the recent 3 years [19–21] showed a clear trend towards CDK4/6 inhibitors instead of chemotherapy. This new era was associated with more local control of the disease with improvement in symptoms and considerable overall survival up to 6 years while kept on under Palbociclib [20]. The results of CDK4/6 inhibitors in the management of eye metastasis confirm the fact that the presence of eye metastasis may not jeopardise survival when treated appropriately.

Recently published cases [20, 22, 23] showed improvement in treatment outcomes secondary to the implementation of CDK4/6 inhibitors in combination with new RTH techniques like Stereotactic Body Radiotherapy (SBRT) [24]. Wiegel *et al* [26] showed that external beam radiotherapy (EBRTH) leads to stabilised or restored vision in up to 86% of patients; the typical dose varies between 20 and 50 Gy [3, 5, 26, 27].

The main challenges were the unavailability of CDK4/6 inhibitors or SBRT and the exhaustion of available hormonal treatment on managing rapidly progressive hormonal resistant metastatic breast cancer. That situation left chemotherapy the only reserve when presenting with eye metastasis with no response. Conformal RTH applied afterward to the persistent huge eye lesion – that was resistant to previous treatment – resulted in disease progression and symptoms worsening.

## Conclusion

With the rising incidence of ocular metastasis due to breast cancer, oncologists should be aware of symptoms and the proper diagnostic modalities. Follow-up on the outcome of treatment is extremely crucial in the absence of guidelines that could help clinical decision. Implementation of CDK4/6 inhibitors and new techniques in RTH in the treatment of breast cancer with eye metastasis opens up new horizons for improving outcomes. We believe that reporting and sharing experiences with these cases is paramount given the relative scarcity of data in this domain.

## Funding sources

This research did not receive any specific grant from funding agencies in the public, commercial or not-for-profit sectors.

## Conflicts of interest

The authors have no conflicts of interest to declare.

## Figures and Tables

**Supplementary Table 1.** Summary of cases reported on the literature on eye metastasis of breast cancer origin.

Case num	Year of publication	Age	Symptom	Histopathology	Molecular subtype	Course of disease	Site of metastasis	Intervention	Outcome	Ref
1	2021	50	Foreign body sensation and exophthalmos in her right eye	IDC with neuroendocrine differentiation	ER+ PR + Her2 - KI67 high Synaptophysin +	Initial presentation and only site of metastasis	Extraoccular muscles and bone destruction	3 cycles nab-paclitaxel followed by Abemaciclib plus Letrazole	Marked reduction of size of mass with improvement in visual acuity.	[1]
2	2021	74	Left eye enophthalmos and ptosis.	IDC	ER+ PR+ Her2 -	Initial presentation and only site of metastasis	Optic nerve	Letrazole for one year then addition of Palbociclib on bone progression	Control for one year on Letrazole then kept on follow-up for 6 years on Palbociclib with preserved visual acuity	[2]
3	2021	76	Enophthalmos and ptosis.	IDC	ER+ PR+ Her2 -	Initial presentation and only site of metastasis	Extraoccular muscles, lacrimal gland and the optic nerve	Letrazole and Palbociclib then SBRT on progression	Progression on hormonal treatment after few months with OS of three years.	[2]
4	2021	33	Blurring vision	N/A	ER+ PR+	Previously diagnosed with EBC one month	Medial rectus muscle and the optic nerve	Treated as an EBC case with surgery followed by adjuvant chemo-radiotherapy and hormonal treatment	Stable disease at three month follow up	[3]
5	2020	54	Blurring vision	IDC	ER- PR- Her2 -	Previously diagnosed with EBC 4 years followed by metastasis to bone and lung	Choroid	Cyperknife 18 GY in single fraction	Total blindness with partial response of the choroid mass	[4]
6	2020	28 cases with median age 55	N/A	IDC 14 ILC 9 Mixed 2 Unknown 3	21 ER+ 17 PR+ 20 HER2 – 3 HER2+	14 Initial presentation 14 following previous diagnosis with breast cancer	11 Extraocular ms 3 Choroid 3 Optic nerve 5 Eyelid 6 Mixed	14 Letrazole 20 radiotheray As first line so treatment	Median OS was 82.1 months	[5]
7	2020	46	Left eye swelling and exophthalmos	IDC	ER + PR+ HER2 -	Initial presentation	Lateral rectus muscle and optic nerve	N/A	N/A	[6]
8	2020	28 cases with median age 50.8 years old	18 decreased visual acuity 16 pain	IDC 14 ILC 13 Papillary 1	26 ER+ 26 PR+ 1 HER2+ 1 Triple positive	12 Initial presentation 16 with previous diagnosis with breast cancer	14 Retina 25 Orbit and muscles 14 Uveal tract	14 Hormonal treatment 14 Chemotherapy 5 radiotherapy 3 surgery 1 intra-ocular Bevacizumab As first line treatment	Median OS after the diagnosis with eye metastasis is 26.4 months	[7]
9	2020	65	Blurring vision	IDC	ER + PR+ HER2 -	Previously diagnosed with EBC 4 years ago	Optic disc and optic nerve	SBRT	Partial response improvement of visual acuity	[8]
10	2019	60	Blurring vision, proptosis	IDC	ER + PR+ HER2 -	Previously diagnosed 3 years for EBC*	Inferior rectus muscle	Fulvestrant and Palbociclib, followed by radiation therapy	Follow up for 6 months, she was free clinically and radiologically	[9]
11	2019	63	Diplopia	IDC	Primary ER/PR+ HER2- Metastasis Triple negative	Previously diagnosed one year before with MBC **	Right medial rectus muscle	Diagnostic biobsy followed by palliative radiotherapy and chemotherapy	Partial improvement	[10]
12	2019	61	orbital fullness with worsening vision and eye pain	NA	ER +	Initial presentation	large ill-defined orbital mass encasing the globe	evisceration	recovered	[11]
13	2019	39	Decrease visual acuity	IDC	ER +	Previously diagnosed 6 years ago for EBC	Mass in the right optic disc with infiltrative optic neuropathy	NA	NA	[12]
14	2018	58	Orbital mass	Infiltrating carcinoma	ER + PR+ HER2 -	Initial presentation	Superior orbital rim	Indoximod plus Docetaxel	NA	[13]
15	2018	46	Diplopia	NA	NA	Previously diagnosed as MBC	Upper left oblique muscle	liposomal Doxorubicin as well as local stereotactic radiotherapy.	showed a gradual improvement of the local symptoms and signs	[14]
16	2017	46	left eye pain	NA	NA	Previously diagnosed 2 years as locally advanced breast cancer	Subretinal mass	NA	NA	[15]
17	2017	56	Diplopia, decreased visual acuity and limited eye movement	IDC	ER +	Initial presentation	Intraconal compartment of the left orbit and invading the adjacent muscles	Excisional biobsy	NA	[16]
18	2015	77	Proptosis	IDC	ER + PR - HER2 -	Previously diagnosed 11 years with EBC	lateral and superior rectus muscle, and eroded the lateral orbital wall and roof	palliative radiotherapy	Died 9 months later	[17]
19	2015	69	Proptosis	IDC	ER + PR - HER2 -	Previously diagnosed one year for EBC	Lacrimal gland	palliative radiotherapy and one cycle chemotherapy	Died 3 month later	[17]
20	2019	56	Decreased visual acuity	IDC	NA	Initial presentation	Choridal	NA	NA	[18]
21	2014	84	Diplopia	NA	NA	Previously diagnosed 14 years EBC	Orbit	NA	NA	[19]
22	2012	48	Blurring vision	IDC	NA	Previously diagnosed 13 years EBC	Choroidal mass	Surgical enucleation	NA	[20]
23	2012	53	Proptosis and diplopia	NA	NA	Initial presentation	Right orbit and skull base	Surgery and palliative radiotherapy	NA	[21]
24	2011	73	Exophthalmus	IDC	ER + PR+ HER2 +	Previous diagnosed 3 ys before as EBC	Suprabulbar tumor mass, with osseous infiltration of the orbital roof	Palliative chemotherapy plus bisphosphonates	Improvement in symptom and continue chemo then hormonal treatment	[22]
25	2011	38	Visual discomfort	NA	NA	Previously diagnosed 3 years with MBC	Bilateral orbital involvement	SPRT	Corrected visual acuity but died 19 month later	[23]
26	2010	66	Swelling and visual disorders	ILC	ER + PR+ HER2 -	Initial presentation	Right peri-orbital soft tissues	Surgery for primary and metastasis then FEC and palliative radiotherapy followed by hormonal treatment	Total resolution	[24]
27	2009	46	Diplopia, proptosis	IDC	ER + PR+ HER2 -	Previously diagnosed 4 years ago as MBC	Intra-orbital extra-bulbar mass	Chemotherapy plus Cyperknife	Gradual improvement in symptoms Free of ocular for 18 months	[25]
28	2009	70	Dimension of vision	NA	NA	Previously diagnosed 9 ys early EBC	Choroid	NA	NA	[26]
29	2008	73	Ptosis and diplopia	ILC	ER + PR +	Previously diagnosed 25 years ago for EBC	Extrinsic muscles and the surrounding tissues	NA	NA	[27]
30	2008	50	Diplopia	ILC	ER+ PR+	Previously diagnosed 5 years for stage III	Bilateral extra-ocular muscles	High dose radiotherapy, hormonal and chemotherapy	NA	[28]
31	2008	66	Unilateral eyelid edema	NA	ER + PR+ HER2 +	Previously diagnosed with stage III	Soft tissue mass in orbit	Trastuzumab, Docetaxel, Tegafur and Cyclophosphamide	Complete response	[29]
32	2008	60	Diminution of vision	IDC	ER + PR+	Previously diagnosed 3 ys for EBC	Uveal tract of the right globe and optic disc	Radiotherapy 44 GY	Complete response	[30]
33	2006	53	Orbital pain and ptosis	ICL	NA	Initial presentation	The medial wall of the left orbit, orbital connective tissue, muscles, and lacrimal gland	NA	NA	[31]
34	2006	58	Swelling in lower eye lid	ILC	ER + PR +	EBC 24 month before	Mass in lower eye lid	NA	NA	[32]
35	2005	75	Binocular diplopia	NA	NA	Previously diagnosed 2 months EBC	Extraocular muscles bilaterally	NA	NA	[33]
36	2005	52	Progressive visual loss and ptosis	IDC	NA	Previously diagnosed as MBC	Bilateral thickening of all extraocular muscle	NA	NA	[34]
37	2004	35	Diplopia and blurred vision	ILC	NA	Previously diagnosed 3 years ago for EBC	Right lateral rectus muscle	Palliative chemotherapy	Died after 10 days	[35]
38	2004	57	Decrease in visual acuity	IDC	Primary ER/PR+ Metastasis HER2 +, ER/PR -	Previously diagnosed 13 ys with EBC	Unilateral choroidal metastasis	Trastuzumab plus Vinolerabin	Complete response	[36]
39	2002	61	Red eye and ptosis	IDC	ER +	Initial presentation	Mass filling the left orbit posteriorly and extending forward in the eyelids	Surgery and local radiotherapy 3000 cGy	NA	[37]
40	2001	40	Loss of vision	NA	NA	Previously diagnosed EBC	Isolated choroid metastasis	Radiotherapy palliative, chemotherapy plus acetazolatime	Clinical and radiological remission	[38]

**Table 1.** Summary of cases reported on the literature on eye metastasis of breast cancer origin.

Number of cases	94 (100%)
Age - Median - Range	56 (33–76)
Histopathology - IDC^a^ - ILC^b^ - Rare histology - Unknown	51 (54.3%) 28 (29.8%) 11 (11.7%) 4 (4.2%)
Immunohistochemical subtype - Hormonal receptors positive - HER2 neu enriched - Triple negative - Not identified	75 (80%) 9 (1%) 19 (2%) 16 (17%)
Treatment modality -Mono-therapy treatment: Surgery alone EBRTH alone^c^ Hormonal alone Chemotherapy alone - Multimodality treatment: Chemotherapy + anti-HER2 neu Hormonal + RT Chemotherapy + RT Surgery + chemotherapy + RTH + hormonal Surgery + chemotherapy+ RTH Surgery + RT RTH + chemotherapy +hormonal Not identified or overlap of data	3 (3.2%) 23 (24.4%) 13 (13.8%) 3 (3.2%) 2 (2.1%) 2 (2.1%) 4 (4.2%) 2 (2.1%) 1 (1.06%) 1 (1.06%) 2 (2.1%) 38 (40.4%)
Primary response Partial/complete response Stable disease Progression Not reported	14 **(**14.8%) 2 (2.1%) 4 (4.2%) 73 (77.6%)

a Infiltrating ductal carcinoma

b Infiltrating lobular carcinoma

c External beam radiotherapy
